# Computational prediction of novel non-coding RNAs in *Arabidopsis thaliana*

**DOI:** 10.1186/1471-2105-10-S1-S36

**Published:** 2009-01-30

**Authors:** Dandan Song, Yang Yang, Bin Yu, Binglian Zheng, Zhidong Deng, Bao-Liang Lu, Xuemei Chen, Tao Jiang

**Affiliations:** 1 Department of Computer Science and Technology, Tsinghua University, Beijing 100084, PR China; 2 Department of Computer Science and Engineering, Shanghai Jiao Tong University, Shanghai 200240, PR China; 3 Department of Botany and Plant Sciences, University of California, Riverside, CA 92521, USA; 4 Laboratory for Computational Biology, Shanghai Center for Systems Biomedicine, Shanghai 200240, PR China; 5 Department of Computer Science and Engineering, University of California, Riverside, CA 92521, USA; 6 Current address: The School of Biological Sciences and the Center for Plant Science Innovation, University of Nebraska, Lincoln, NE 68588, USA

## Abstract

**Background:**

Non-coding RNA (ncRNA) genes do not encode proteins but produce functional RNA molecules that play crucial roles in many key biological processes. Recent genome-wide transcriptional profiling studies using tiling arrays in organisms such as human and *Arabidopsis *have revealed a great number of transcripts, a large portion of which have little or no capability to encode proteins. This unexpected finding suggests that the currently known repertoire of ncRNAs may only represent a small fraction of ncRNAs of the organisms. Thus, efficient and effective prediction of ncRNAs has become an important task in bioinformatics in recent years. Among the available computational methods, the comparative genomic approach seems to be the most powerful to detect ncRNAs. The recent completion of the sequencing of several major plant genomes has made the approach possible for plants.

**Results:**

We have developed a pipeline to predict novel ncRNAs in the *Arabidopsis *(*Arabidopsis thaliana*) genome. It starts by comparing the expressed intergenic regions of *Arabidopsis *as provided in two whole-genome high-density oligo-probe arrays from the literature with the intergenic nucleotide sequences of all completely sequenced plant genomes including rice (*Oryza sativa*), poplar (*Populus trichocarpa*), grape (*Vitis vinifera*), and papaya (*Carica papaya*). By using multiple sequence alignment, a popular ncRNA prediction program (RNAz), wet-bench experimental validation, protein-coding potential analysis, and stringent screening against various ncRNA databases, the pipeline resulted in 16 families of novel ncRNAs (with a total of 21 ncRNAs).

**Conclusion:**

In this paper, we undertake a genome-wide search for novel ncRNAs in the genome of *Arabidopsis *by a comparative genomics approach. The identified novel ncRNAs are evolutionarily conserved between *Arabidopsis *and other recently sequenced plants, and may conduct interesting novel biological functions.

## Background

Since the genome of *Arabidopsis thaliana *has been sequenced, a wide range of plant-specific genes as well as genes with homologs in non-plant species have been identified. The annotated genes encode either proteins or non-coding RNAs (ncRNAs), *i.e.*, RNAs that function by means other than directing the production of proteins. Besides the known gene regions, there are still large areas in the *Arabidopsis *genome that remain unannotated. Within the unannotated regions may reside as yet undiscovered ncRNAs.

ncRNAs are known to play crucial roles in many key biological processes as structural components of ribonucleoprotein complexes, as sequence-specific guides, or as enzymes. Classic examples of ncRNAs include the RNA component of the signal recognition particle (SRP_RNA) that directs subcellular protein transport, small nuclear RNAs (snRNAs) that act in splicing, tRNAs and rRNAs that function in translation, small nucleolar RNAs (snoRNAs) that direct RNA modifications, the telomerase RNA that acts in telomere maintenance, and the RNA component in PTEFb involved in transcription elongation. In recent years, small RNAs consisting of 21 to 30 nucleotides have been uncovered in plants and animals and found to serve as sequence-specific guides in transcriptional and post-transcriptional control of gene expression.

While the above-mentioned ncRNAs are found in diverse organisms such as plants and animals, some lineage-specific ncRNAs with key biological functions have also been uncovered. One example is the Xist RNA that is crucial in the silencing of the inactive X chromosome in mammals. Xist is involved in recruiting the polycomb group proteins (PcG), which lead to trimethylation of histone H3 lysine 27 (H3K27) to result in heterochromatin formation [1]. Long ncRNAs mediating epigenetic chromatin modifications may be a general theme in metazoans. A recent study identified numerous ncRNAs from the HOX loci in humans and showed that an ncRNA named HOTAIR represses the transcription of the target HOXD locus in trans through H3K27 trimethylation [2].

Recent genome-wide transcriptional profiling studies with tiling arrays in human and in *Arabidopsis *revealed that an unexpectedly large portion of the genome gives rise to transcripts [3-5]. Although not all transcripts may prove to be functional, this unexpected finding suggests that the currently known repertoire of ncRNAs may only represent a small fraction of ncRNAs of the organisms. In fact, it is not surprising that our knowledge of ncRNAs is quite limited. The lack of well-defined sequence characteristics of ncRNAs (as opposed to those of protein-coding mRNAs) makes it hard to infer ncRNAs directly from genomic sequences. As a result, the annotation of ncRNA genes has lagged behind that of protein-coding genes. Moreover, the lack of homology between ncRNAs from plants and those from other types of organisms prevents homology-based ncRNA discovery. Studies on plant ncRNAs have mainly been focused on microRNAs (miRNAs) and several kinds of structural RNAs. Little is known about ncRNAs other than miRNAs and the ones with homologs in animals or yeast. A study in 2001 examined expressed sequence tags (ESTs) from *Arabidopsis *for the lack of long opening reading frames and identified 19 potential ncRNAs [6]. None of them have homologs in animals, suggesting that plants have their own complement of ncRNAs in addition to the ones that are conserved in all eukaryotes [6,7].

The identification of ncRNAs has been pursued extensively in the bioinformatics community in recent years. A number of ncRNA databases have been created, such as Rfam [8] that contains a large number of ncRNA families, miRBase [9] for microRNAs, and the plant snoRNA database [10]. Since many ncRNAs have conserved secondary structures, most of the computational methods for predicting ncRNAs make use of secondary structure information. Covariance model [11] and context-sensitive HMMs [12] are two popular methods for ncRNA identification. But, they search for ncRNAs based on pre-defined models of known ncRNA families and are thus not designed to find novel ncRNAs. There are other methods for detecting ncRNAs within a single genome that could potentially find novel ncRNAs. Will *et al. *inferred ncRNA families by means of genome-scale structure-based clustering in the *Ciona intestinalis *genome [13]. They found some clusters that could represent candidates of novel ncRNA classes. Adai *et al. *predicted miRNAs using a target-based method that can find specific miRNAs unique in *Arabidopsis *[14]. Compared with the methods based on a single genome, homology-based prediction of ncRNAs using multiple genomes is more widely used, because many important ncRNAs are expected to be conserved in certain genomes. The comparative genomics approach has been successfully applied to identify novel ncRNAs in several groups of organisms including animals [5,15], yeast [16], and bacteria [17-19]. However, the approach has not been applied to plants on a genome scale before because the number of completely sequenced plant genomes was limited.

In addition to *Arabidopsis *and rice (*Oryza sativa*), three other plant genomes have been completely sequenced recently including poplar (*Populus trichocarpa*), grape (*Vitis vinifera*), and papaya (*Carica papaya*). This new rapid increase of information makes it possible to conduct a genome-wide search for plant ncRNAs using the comparative genomics approach. In this paper, we develop a computational pipeline to predict novel ncRNAs in *Arabidopsis *following the comparative genomics approach. We combine a number of information sources including tiling array expression data, sequence homology between *Arabidopsis *and other recently sequenced plant genomes, and RNA secondary structure stability and conservation (as used in RNAz, which is a popular ncRNA prediction program). The putative ncRNAs are then validated using wet-bench experiments (for expression), compared with the known ncRNAs in various ncRNA databases (for novelty), and examined for their protein-coding potentials. The analysis resulted in a total of 16 families of novel ncRNAs (consisting of 21 ncRNAs). These ncRNAs may possess novel functions and play roles in some important biological processes in plants.

## Results

### Prediction of ncRNAs in *Arabidopsis*

At the time when the project was initiated, only the complete genomes of *Arabidopsis*, rice and poplar were available. Our initial analysis was thus based on this triplet of genomes, abbreviated as ARP. Later on, the complete grape genome became available, and we decided to analyze the triplet *Arabidopsis*, grape and poplar (AGP) and then took the intersection of its result with that of ARP. An advantage of this approach (as opposed to combining all four genomes together) is that it allows us to also identify ncRNAs that are conserved in a triplet but not in all four genomes. During the preparation of this manuscript, the papaya genome was completed. Since poplar is common to both triplets ARP and AGP, we analyzed another triplet *Arabidopsis*, papaya and poplar (APP) and used its result to double check those of ARP and AGP. Note that, since papaya is the closest to *Arabidopsis *evolutionarily among the four genomes (followed by grape and poplar, with rice being the farthest), the putative ncRNAs from ARP and AGP are likely to be also in the result of APP.

Each above triplet of plant genomes is analyzed by our computational pipeline independently as follows. The transcribed non-coding regions (TNRs) of *Arabidopsis *are collected from tiling array experiments and their counterparts in the intergenic regions of each of the other two plant genomes in the triplet are searched for separately using BLASTN. The search results in many local pairwise alignments (high scoring pairs, or HSPs). Two HSPs overlap on the *Arabidopsis *genome are combined and extended to make a multiple sequence alignment, which is then refined using ClustalW. Finally, the multiple alignments are fed to RNAz to predict candidate ncRNAs. The details of the pipeline will be given in the Materials and Methods section.

We conducted two predictions, based on two different sources of tiling array data. The result of the first prediction, called group I ncRNAs, was obtained using the whole genome tiling array data reported in Yamada *et al. *[3] and the result of the second prediction, called group II ncRNAs, was obtained using the supplementary tiling array data when certain subunits of the exosome were depleted [20]. The numbers of predicted ncRNAs in groups I and II are listed in Tables 1 and 2, respectively.

**Table 1:** Group I result. ARGP stands for the intersection of ARP and AGP.

	Chromosome					
	1	2	3	4	5	Total
ARP	54	68	107	17	51	297
AGP	106	111	639	48	253	1157
ARGP	38	36	68	7	30	179

Filter 1	4	13	10	0	11	38
Filter 2	4	13	9	0	8	34
Filter 3	4	9	5	0	5	27

The row of Filter 1 shows the numbers of predicted ncRNAs that exist in TAIR8 intergenic regions. The row of Filter 2 shows the numbers of putative ncRNAs that do not exist in ncRNA databases. The row of Filter 3 shows the numbers of candidate novel ncRNAs that cannot be classified by Rfam.

**Table 2:** Group II result. The three filtration steps are the same as in Table 1.

	Chromosome					
	1	2	3	4	5	Total
rrp4 ARP	17	2	28	40	33	120
AGP	19	2	495	54	235	805
AGRP	14	2	20	25	24	85
rrp41 ARP	8	0	28	12	14	62
AGP	11	0	490	22	225	748
AGRP	6	0	20	6	14	46
csl42 ARP	6	2	32	12	14	66
AGP	9	2	482	25	224	742
AGRP	6	2	22	2	13	45
Total ARP	17	4	32	43	41	137
AGP	19	4	500	57	239	819
AGRP	14	4	22	27	33	100

Filter 1	6	2	22	12	33	75
Filter 2	5	1	22	10	30	68
Filter 3	0	0	4	0	18	22

In group I, a total of 297 *Arabidopsis *ncRNAs were predicted for ARP and 1157 ncRNAs were predicted for AGP. The intersection of these two results yields 179 putative ncRNAs, which are more likely to be true ncRNAs than those predicted from each individual triplet. The distribution of these numbers in the five chromosomes of *Arabidopsis *is shown in Table 1. In the tiling array experiment used in group II, three exosome subunits, RRP4, RRP41 and CSL42, were depleted independently, which gave rise to three different ncRNA predictions. We list them separately in Table 2. Since the prediction results of the three depletion experiments overlap, we also list the total numbers of distinct putative ncRNAs from all three depletion experiments.

### Screening against known genes and ncRNAs

This part of analysis aims to filter out the predicted ncRNA candidates that match annotated genes in TAIR8 (the most up-to-date annotation of *Arabidopsis*), as well as known ncRNAs contained in public databases such as miRBase and EMBL. The remaining predictions are then classified using the INFERNAL software with Rfam models to see if they belong to any known ncRNA families.

#### Matching the predictions to TAIR8 annotations

The tiling array experiments used in group I and group II results were based on the genomic sequence and annotations of TAIR3 and TAIR6, respectively. However, the *Arabidopsis *genome has been updated and re-annotated since then. Thus, our predicted ncRNAs were matched to the latest version of the *Arabidopsis *genome annotation (TAIR8 [21]) to confirm their existence on the genome and exclude the genes that been annotated since the old versions.

For group I, 165 among the 179 predicted ncRNAs were found to exist (exactly) in the new version of the genome, among which 37 are still located in intergenic regions. The predictions that overlap with annotated genes were checked in detail. 60 sequences located on the same strand as their matching annotations include 11 tRNAs, 2 rRNAs, 1 ncRNA, 4 misc_RNAs, 1 CDS, 1 mRNA (the predicted sequence overlaps after the last CDS segment), and 40 misc_features (including 35 transposable element genes and 5 pseudogenes). Since the predicted sequence that overlaps with a known CDS only overlaps at its end positions, it was not excluded here but analyzed later on in the wet-bench experiments (named SEQ5a). Thus, a total of 38 putative ncRNAs are kept and passed on to the next filtration step.

For group II, among the 100 predicted segments, 25 were found to overlap with current annotations in TAIR8, in which 13 are located on the same strand including 6 tRNAs and 7 misc_features (more specifically, transposable elements). These sequences were thus eliminated.

#### Matching the predictions to known ncRNA databases

Two public databases of ncRNAs were used to exclude known ncRNAs from our predictions. One is miRBase, which contains published miRNA sequences and annotations [22,23]. Its ath.gff file containing chromosomal coordinates of *Arabidopsis *microRNAs with version 11.0 and update date of April 11, 2008 was downloaded and applied. The other ncRNA database was obtained from EMBL [25]. After downloading its Rel. 83, Version 37 of the *Arabidopsis *genome annotation, all tRNAs annotated by tRNAScan-SE-1.23 and ncRNAs annotated by rfam_scan.pl were extracted. Our prediction results were screened against these known ncRNAs. Among the 38 remaining candidates in group I, 4 were found in the EMBL database, including 3 snRNAs and 1 SRP_RNA. Among the 75 candidates in group II, 7 were found in the EMBL database, including 5 snRNAs and 2 SRP_RNAs. None of our predictions were found in miRBase. The 4+7 sequences are deemed as known ncRNAs and removed from further consideration. In addition, we have also compared our result against the recently found mRNA-like non-coding RNAs (mlncRNAs) in Arabidopsis [26]. None of our predictions match the mlncRNAs.

#### Classifying the putative ncRNAs using Rfam

For the predicted ncRNAs that survived in the last two steps of filtration, the INFERNAL software package [27] (version 0.72) was used to classify them into families according to the Rfam database [8]. All the 607 families in version 8.1 of Rfam were applied to set up the models.

Among the 34 remaining sequences in group I, INFERNAL predicted 4 5s-rRNAs, 3 microRNAs, 2 snRNAs (more specifically, spliceosomal RNAs), and 2 Group II catalytic introns. As we are interested in novel ncRNAs, the 23 predictions that were not classified by INFERNAL into any Rfam families were kept. However, it should also be noted that these classification results do not have wet-bench experimental support and may thus be erroneous. Therefore, we analyzed the classified predictions in detail and selected 4 more sequences that unlikely belong to known ncRNA families (2 Group II catalytic introns and 2 microRNAs) for further analysis. This resulted in a total of 27 candidates for novel ncRNAs.

For group II, a total of 46 predictions were classified into known Rfam families. Including 33 rRNAs, 11 spliceosomal snRNAs, 1 CD-box snoRNA, and 1 SRP_RNA, leaving us with 22 candidates for novel ncRNAs for further analysis.

The above novel ncRNA candidates are listed in Tables 3 and 4. In the tables, the sequences are named with suffixes a/b if they have sequence similarity at least 90%, or suffixes W/C if they are located on the opposite strands of the same locus.

**Table 3:** Detailed information of the novel ncRNA candidates in group I.

			Genome Location			
Name	Chr.	Intergenic Region	Start	End	Strand	Len.
SEQ1	1	AT1G28750–AT1G28770	10091146	10091292	W	147
SEQ2	1	AT1G30972–AT1G30974	11046502	11046670	W	169
SEQ3^‡^	1	AT1G43620–AT1G43630	16433517	16433593	W	77
SEQ4	1	AT1G54890–AT1G54905	20470604	20470753	C	150
SEQ5a^†^	2	AT2G01020–AT2G01022	6084	6646	C	563
SEQ6a^†^	2	AT2G01020–AT2G01022	6708	7257	C	550
SEQ7a^#,†^	2	AT2G01020–AT2G01022	7288	8699	C	1412
SEQ8a^†^	2	AT2G01020–AT2G01022	8889	9367	C	479
SEQ9W	2	AT2G07590–AT2G07600	3187035	3187217	W	183
SEQ9C*,^#^	2	AT2G07590–AT2G07600	3187035	3187217	C	183
SEQ10W	2	AT2G07689–AT2G07691	3338151	3338560	W	410
SEQ10C	2	AT2G07689–AT2G07691	3338151	3338560	C	410
SEQ11C	2	AT2G07732–AT2G07733	3469569	3469970	C	402
SEQ11W*	2	AT2G07732–AT2G07733	3469611	3470020	W	410
SEQ12W	2	AT2G09880–AT2G09890	3747858	3748031	W	174
SEQ12C^*,‡^	2	AT2G09880–AT2G09890	3747858	3748031	C	174
SEQ13^‡^	2	AT2G12420–AT2G12430	5036416	5036560	C	145
SEQ5b^†^	3	AT3G41979–AT3G42050	14211041	14211603	C	563
SEQ6b^†^	3	AT3G41979–AT3G42050	14211665	14212214	C	550
SEQ7b^#,†^	3	AT3G41979–AT3G42050	14212350	14213656	C	1307
SEQ8b^†^	3	AT3G41979–AT3G42050	14213846	14214324	C	479
SEQ14^♭^	3	AT3G42803–AT3G42806	14923566	14923707	W	142
SEQ15	5	AT5G09960–AT5G09970	3111082	3111016	W	67
SEQ16	5	AT5G29805–AT5G29890	11327943	11328007	W	65
SEQ17	5	AT5G32410–AT5G32420	12042758	12042828	C	71
SEQ18a	5	AT5G34358–AT5G34376	12842957	12843077	W	121
SEQ18b	5	AT5G34412–AT5G34431	12885676	12885796	W	121

The three sequences marked by * are not expressed in the bi-directional RT-PCR analysis of the inflorescence tissue. The three sequences marked by # may code for proteins. The sequences marked by † were annotated as LSU-rRNA Ath by RepeatMasker. The sequences marked by ‡ were annotated as LTR by RepeatMasker. The sequences marked by ♭ were annotated as DNA transposons by RepeatMasker.

**Table 4:** Detailed information of the novel ncRNA candidates in group II. The legends have the same meaning of those in Table 3.

			Genome Location			
Name	Chr.	Intergenic Region	Start	End	Strand	Len.
SEQ19a^‡^	3	At3g33055–At3g33058	13598866	13598930	W	65
SEQ19b^‡^	3	At3g33055–At3g33058	13601377	13601440	W	64
SEQ20a^†^	3	At3g41979–At3g42050	14213822	14214274	C	453
SEQ20b^†^	3	At3g41979–At3g42050	14213822	14214274	W	453
SEQ21	5	At5g29805–At5g29890	11327943	11328007	W	65
SEQ22C^‡^	5	At5g34358–At5g34376	12828009	12828088	C	80
SEQ22W	5	At5g34358–At5g34376	12828009	12828088	W	80
SEQ23aC^‡^	5	At5g34358–At5g34376	12833485	12833557	C	73
SEQ23aW	5	At5g34358–At5g34376	12833485	12833557	W	73
SEQ23bC^‡^	5	At5g34358–At5g34376	12834229	12834301	C	73
SEQ23bW	5	At5g34358–At5g34376	12834229	12834301	W	73
SEQ24a^‡^	5	At5g34358–At5g34376	12836733	12836797	C	65
SEQ23c^‡^	5	At5g34358–At5g34376	12841209	12841281	C	73
SEQ23dC^‡^	5	At5g34358–At5g34376	12841964	12842030	C	67
SEQ23dW	5	At5g34358–At5g34376	12841964	12842030	W	67
SEQ25a	5	At5g34358–At5g34376	12842908	12843077	W	170
SEQ24b^‡^	5	At5g34412–At5g34431	12875464	12875528	C	65
SEQ24c^‡^	5	At5g34412–At5g34431	12879452	12879516	C	65
SEQ23e^‡^	5	At5g34412–At5g34431	12882930	12882999	C	70
SEQ23fC^‡^	5	At5g34412–At5g34431	12883928	12884000	C	73
SEQ23fW	5	At5g34412–At5g34431	12883928	12884000	W	73
SEQ25b	5	At5g34412–At5g34431	12885627	12885796	W	170

### Experimental validation

**Figure 1:**
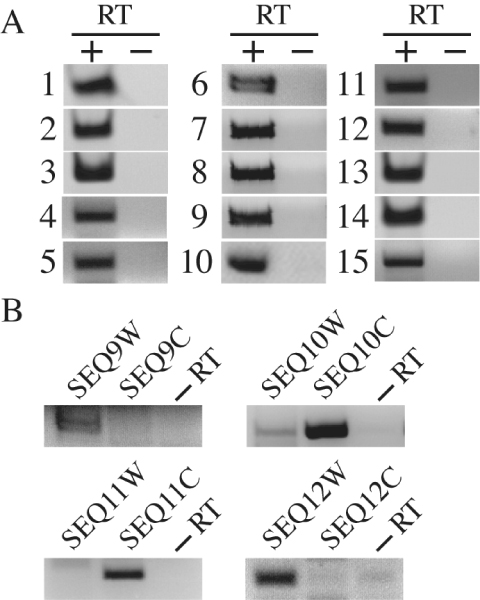
**RT-PCR analysis of the novel ncRNA candidates in group I**. (A) ncRNAs predicted to be transcribed in only one direction. Reverse transcription was performed using Oligo dT primers. 1: SEQ1; 2: SEQ2; 3: SEQ3; 4: SEQ4; 5: SEQ13; 6: SEQ5a or SEQ5b; 7: SEQ6a or SEQ6b; 8: SEQ7a or SEQ7b; 9: SEQ8a or SEQ8b; 10: SEQ5a combined with SEQ6a or SEQ5b combined with SEQ6b; 11: SEQ14; 12: SEQ15; 13: SEQ16; 14: SEQ17; 15: SEQ18a or SEQ18b. (B) ncRNAs predicted to be transcribed in both directions. Reverse transcription was performed using primers complement to the predicted ncRNAs. Here, -RT denotes negative control.

To confirm that the loci containing the above candidate novel ncRNAs in group I are actually transcribed into polyadenylated RNAs, we detected RNAs from these loci by reverse-transcription PCR. Total RNA was isolated from inflorescence tissue and subjected to reverse transcription (RT) using oligo dT primers. PCR was carried out on the RT or control reactions using gene specific primers corresponding to the 19 loci in Table 3 that were predicted to be transcribed in only one direction. Indeed, RNA was detected at each of the loci. (See Fig. 1A). Notice that, some of the sequences (*i.e.*, SEQ5a and SEQ5b; SEQ6a and SEQ6b; SEQ7a and SEQ7b; SEQ8a and SEQ8b; SEQ18a and SEQ18b) are so similar that they cannot be distinguished in the experiment. Additionally, there are sequences (SEQ5(a,b) with SEQ6(a,b)) that located in the same intergenic region with short gaps, the PCR experiments are also conducted on their combined sequences. Four other loci were predicted to generate RNAs in both directions. We tested for RNAs from both directions by using strand-specific primers in the RT reactions followed by PCR. One of the four loci (SEQ10W/C) gave rise to RNAs from both strands while the other three produced RNAs only from one strand (Fig. 1B, also marked in Table 3). Thus, the transcription of 24 of the above novel ncRNA candidates are experimentally verified. However, as our RT-PCR experiments were only based on the inflorescence tissue, the 3 RNAs that were not expressed in the experiments still have the possibility to be expressed in other tissues.

For the novel ncRNA candidates from group II, we were unable to experimentally verify their expression since we do not have the means to deplete exosomes.

### Comparison with the prediction result from APP

After the genome of Papaya was published, the same computational pipeline was applied on the triplet APP. In group I, 940 ncRNAs were predicted by our pipeline. Among the 179 ncRNAs predicted for both ARP and AGP, 153 (85.47%) can be found in the APP result. Moreover, 26 of the 27 novel ncRNAs in Table 3 (96.30%) were found in the APP result, except for SEQ13. In group II, 706 RNAs were predicted by our pipeline for APP. Among the 100 ncRNAs predicted for both ARP and AGP, 89 (89.00%) can be found in the APP result. Similarly, 21 (95.45%) of the 22 novel ncRNA candidates in Table 4 were found in the the APP result, except for SEQ22C. This high level of containment is consistent with the fact that papaya is evolutionarily the closest to *Arabidopsis *among the four plant genomes, and our candidates for novel ncRNAs are conserved in all five plant genomes.

### Further analysis

After experimentally verifying the expression of the novel ncRNA candidates (in group I), we studied the possibility that some of these genes actually code for proteins. We also checked if these genes contain small RNAs or are repeats or transposons.

#### Protein coding potential

To make sure that our predicted novel ncRNAs do not code for proteins, we analyzed the protein-coding potential for each of the novel ncRNA candidates.

First, the novel ncRNA candidates were screened by NCBI ORF Finder [28] to detect possible ORFs. Some candidates were predicted to contain one or more ORFs. The ORFs whose lengths are less than 100 nt were ignored. Then all the ORFs were searched for homologs using BLASTP against the non-redundant protein sequence database (nr) at NCBI. We also examined how the nucleotide substitution rate varies among the positions within a codon in the ORFs. This information could be used to discriminate protein coding and non-coding sequences. For our purpose, the null hypothesis is that conservation of a nucleotide in an ncRNA is independent of the reading frame, *i.e.*, the substitution rate has a uniform distribution across all positions within a codon.

The above analysis classified the candidates into three types. Type I sequences have no ORFs, and are thus most likely non-coding. Type II sequences have ORFs and match some known proteins with e-value less than 10^-6^. For this type, we further looked at the results of BLASTP and checked whether their matches to known proteins are exact or partial. Type III sequences have ORFs, but they have no homologs in the protein database nr, or have only homologs with unknown or hypothetical proteins. For each sequence of the last type, we determined its protein coding potential by checking the nucleotide substitution rate at each of the three positions within a codon as follows. First, we search for the sequence in the other four plant genomes using TBLASTX. The e-value cutoff was set as 10^-6^. Then the ORFs conserved in at least 3 other plants were retrieved and a multiple alignment was constructed. To estimate the significance of deviation from the above null hypothesis, we calculated the G-statistics [16,29] of the three sets of columns of the aligned ORFs (corresponding to the three positions within a codon).

Among the 27 novel ncRNA candidates in group I, 11 belong to Type I, 3 belong to Type II, and the other 13 belong to Type III. For the three Type II candidates, an ORF of SEQ7(a,b) was found to match a transcription factor in *Brugia malayi *nearly over its entire length with e-value 10^-22^, but the protein has not been verified by experiments. An ORF of SEQ9C was found to match the protein Ycf1 in *Cercidiphyllum japonicum *with e-value 10^-20^, which makes SEQ9C most likely a protein-coding gene. These three sequences will be excluded from our final candidate set. The G-statistic scores of the 13 Type III candidates are all below 5.99. Since scores greater than 5.99 indicate significant deviation from the null hypothesis at the 0.05 confidence level with 2 degrees of freedom, we have not observed significant variation of nucleotide substitution rate across the three positions within a codon. Therefore, except for 3 candidates, 24 candidates in group I are likely non-coding.

Similar analysis was performed on the 22 group II candidates. Only one sequence was predicted to have an ORF. But, as this ORF is not conserved in other plant genomes, all the 22 ncRNAs are considered as non-coding.

#### Containment of small RNAs

As the first attempt to explore the functionality of our predicted ncRNAs, we also examined the presence of known small RNAs matching to the predicted sequences. The file smallRNA_sequences. fasta from the ASRP database [30] containing all the small RNA sequences (totally 206077 small RNAs) was downloaded and used as the reference database. The occurrence numbers of the exact nucleotide sequences of the small RNAs in our predicted ncRNA segments were recorded.

For group I, among the novel ncRNA candidates listed in Table 3, four contain no known small RNAs at all (SEQ1, SEQ9W, SEQ9C, and SEQ14). On the other hand, eight candidates contain hundreds of small RNAs (*i.e. *SEQ(5–8)(a,b)). The numbers of small RNAs contained in the other 15 candidates vary between 1 and 46. For group II, two candidates (*i.e.*, SEQ20(a,b)) listed in Table 4 contain 784 small RNAs on both strands. The other 20 candidates contain small RNAs between 33 to 134 each.

#### Identification of repeats and transposons

We also checked whether the novel ncRNA candidates could represent repeats in the *Arabidopsis *genome. A gene is defined to have repeats if it has more than 5 homologous segments including itself on the genome with sequence identities more than 70%. Among the 27 candidates in group I, 6 have repeats (SEQ3, SEQ13, SEQ16, SEQ17, and SEQ18(a,b)). 18 of the group II candidates have repeats (except for SEQ20(a,b), SEQ25(a,b)).

RepeatMasker is used to check if our novel ncRNA candidates could be transposons. The parameters used are: WUBlast search engine, default Speed/Sensitivity, with *Arabidopsis *DNA source. For group I, 3 of the 27 candidates were found to match Retroelements (LTR elements) and another was found to match a DNA transposon. Moreover, the sequences of SEQ(5–8)(a,b) were found to be rRNAs by RepeatMasker. Recall that the same sequences were also found to contain many small RNAs in the previous step. Among the 22 novel ncRNA candidates in group II, 12 matched LTR elements. The 2 sequences (*i.e. *SEQ20(a,b)) that are rich in small RNAs also matched rRNAs. The above matched sequences are marked in Tables 3 and 4. These marked sequences are excluded from the final prediction result.

## Discussion

A few of the predicted novel ncRNA loci were found to contain numerous small RNAs. It is very likely that the candidate novel ncRNAs at these loci are precursors to endogenous small RNAs. The small RNAs are most likely endogenous siRNAs as opposed to microRNAs since there are multiple small RNAs located on both strands of the loci. Endogenous siRNAs are known to cause local transcriptional gene silencing through DNA or histone methylation, Therefore, one role of these ncRNA loci could be to define local heterochromatin to affect nearby gene expression or to prevent transcriptional read-through from nearby genes.

There are a number of predicted novel ncRNA loci that are unlikely to be transposable elements or to generate small RNAs. These loci likely contain ncRNAs with novel biological functions, and will be further studied in the future. Known ncRNAs have been shown to act in numerous cellular processes, such as chromatin modifications, transcription, RNA processing, RNA modifications, translation, and regulation of gene expression at the posttranscriptional levels. Therefore, the predicted ncRNAs can potentially act in any of these or other processes. The temporal and spatial expression patterns of these genes and the analysis of T-DNA insertion mutants in them may provide hints to their biological functions.

## Conclusion

We have conducted a de novo computational prediction of novel ncRNAs in the whole genome of *Arabidopsis*. The prediction results were validated and refined using wet-bench experiments and various public databases and analysis tools with stringent criteria. Finally, for group I data, 13 novel ncRNAs were identified, which can be divided into 12 clusters where each cluster contains sequences with similarity of more than 90% and can be viewed as a gene family. Similarly, for group II data, 8 novel ncRNAs were identified that form 4 gene clusters. That is, a total of 21 loci were predicted to contain 16 families of novel ncRNA genes.

## Materials and methods

### Data sources

To obtain reliable ncRNA predictions, we used two high-resolution genome tiling arrays on *Arabidopsis *and four other completely sequenced plant genomes. The data sources are described below.

#### Expressed intergenic regions of *Arabidopsis*

Yamada *et al. *[3] produced high-density oligonucleotide arrays for the entire genome of *Arabidopsis*. According to their Gene Expression Omnibus analysis, two categories of data were adopted in our research, namely Expression of Integenic Clusters and Expression in Intergenic Regions. Intergenic clusters are novel (unannotated) genes detected by aligning *Arabidopsis *cDNAs/ESTs to the *Arabidopsis *genome. Intergenic regions are regions containing neither annotated genes nor intergenic clusters. Considering that the intergenic clusters may also contain RNA genes, we took both kinds of expression regions into consideration.

In addition to this tiling array data, we also adopted another high-resolution genome tiling array data published recently by Chekanova *et al. *[20]. Many new transcripts of *Arabidopsis *were revealed by depleting the subunits of exosome substrates, RRP4, RRP41 and CSL4, respectively. Thus, numerous mRNAs, miRNAs and other types of ncRNAs were implicated. The paper provides a list of regions where novel ncRNAs may reside. Many regions are quite short, and some of them are located near each other.

We extracted these candidate ncRNA regions and concatenated those that are located in the same intergenic region to form the second tiling array data.

#### Intergenic regions of the other plant genomes

• **Rice **The International Rice Genome Sequencing Project was completed in 2002 [31]. The intergenic region sequences were downloaded from annotation version 5.0 [32], including all the 12 chromosomes.

• **Poplar **The draft genome of poplar was reported in 2006 [33]. The poplar genome consists 19 chromosomes, which is four times larger than the genome of *Arabidopsis*. We eliminated the annotated gene regions from the poplar genome release 1.1 and used the remaining regions of the genome for homology search.

• **Grape **A high-quality draft of the genome sequence of grape was released in 2007 [34]. According to the annotation v1, we extracted intergenic sequences by exluding the regions annotated as genes, mRNAs, CDSs or UTRs from the whole genome.

• **Papaya **Recently, Ming *et al. *published a draft genome of the transgenic tropical fruit tree papaya [35]. We got the genome data from the authors, including the scaffolds and predicted gene positions for all nine pairs of chromosomes. Similarly, we obtained intergenic regions by removing annotated gene sequences. For the scaffolds that have no annotation, we simply used their entire sequences.

### RT-PCR methods

Total RNA was extracted with Tri-reagent (Molecular research center) from inflorescences followed by DNase I (Promega) treatment. Reverse transcription was performed with Superscript II reverse transcriptase (Invitrogen) using oligo dT primer or primers complementary to the predicted ncRNAs. After reverse transcription, PCR reactions were performed using ncRNA specific primers, and PCR products were separated on a 3.2% agarose gel and visualized by ethidium bromide staining.

### Computational prediction pipeline

**Figure 2:**
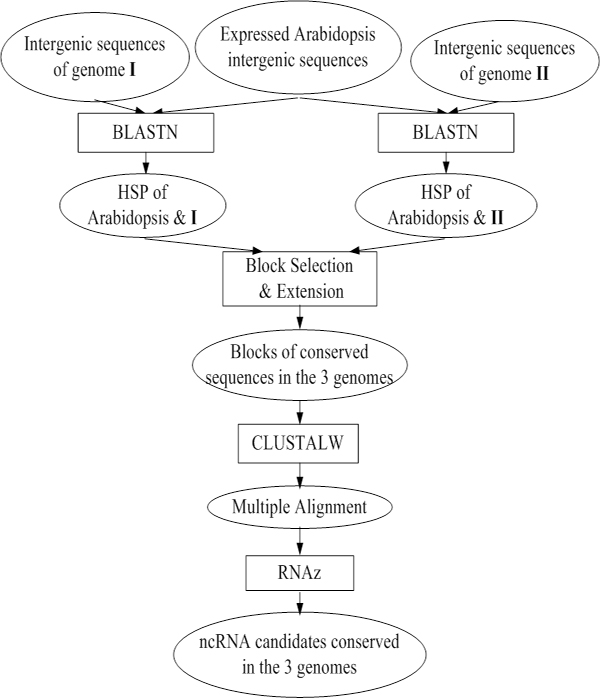
**Flowchart of the computational prediction pipeline**. In the flowchart, a rectangle represents an action and an oval represents a dataset.

As mentioned in the Results section, the five plant genomes were grouped into three triplets, ARP, AGP and APP. Each triplet consists of expressed intergenic sequences of *Arabidopsis *and intergenic sequences of two other genomes. Fig. 2 shows a flowchart of the computational prediction procedure for a triplet. The key steps of the pipeline are explained below.

#### Construction of conserved blocks

The pipeline starts from the transcribed non-coding regions of *Arabidopsis *as given in the tiling array data, and attempts to find their counterparts in each of the other two genomes by using BLASTN. The e-value cutoff is set to 0.01. This cutoff is not stringent. The reason we set a relatively low criterion on sequence conservation is based on the consideration that ncRNAs are not very well conserved in sequence although they tend to be more conserved in secondary structure. Moreover, Zhang *et al. *conducted some controlled experiments on human and mouse and concluded that the e-value cutoff of 0.01 is optimal for identifying homologs since it provides the greatest separation between real homologs and negative controls [5]. 

Two sets of HSPs are obtained from the BLASTN search, namely HSPs of *Arabidopsis *& genome I and HSPs of *Arabidopsis *& genome II. These HSPs are combined to form blocks as follows. If an HSP from *Arabidopsis *& genome I overlaps with another HSP of *Arabidopsis *& genome II on the *Arabidopsis *genome, they are combined into a 3-sequence alignment in a straightforward way. The resultant 3-sequence alignment is called a block.

Obviously, the lengths of the three sequences may be very different if the two original HSPs overlap by a small fraction. Since the *Arabidopsis *sequence in such a 3-sequence alignment is usually the longest, we extend the other two sequences to the length of the *Arabidopsis *sequence. Moreover, some of the HSPs could be very short. Blocks constructed from these HSPs might not provide any signals since they are too short to predict an entire RNA secondary structure. Therefore, we perform a second step of extension. Basically, each sequence in a block could be extended on both sides by up to 50 nucleotides according to our statistics on the average length of ncRNAs in Rfam. It should be noted that, not every sequence can be extended as long as we wish because the extension may get into known genes or reach the end of a chromosome or scaffold. To ensure high-quality blocks that contain sequences with approximately equal lengths, we calculate the minimum number of nucleotides on each side of the block by which every sequences could be extended, and do the extension accordingly. Therefore, the involved *Arabidopsis *sequence could actually be truncated rather than extended in this case.

Both original and extended blocks are considered in the subsequent steps of our pipeline. If a consistent classification is obtained by RNAz on both the original and extended blocks (the extended one contains the original one), we use the extended one for further analysis.

Because the input multiple alignment is crucial to the performance of RNAz, the multiple alignment of the three sequences in a block is refined by ClustalW [36] before it is fed to RNAz.

#### ncRNA prediction

RNAz [37] detects evolutionary conserved and thermodynamically stable RNA secondary structures in the input multiple alignment, and uses a support vector machine to determine whether or not the input multiple alignment could represent a RNA. It has been demonstrated as a very efficient tool for identifying candidate ncRNAs with high sensitivity and specificity [7,38,39].

Because RNAz requires the input alignment to be at most 400 columns, and most of our extended blocks exceed this threshold, we divide a long alignment of the extended blocks into overlapping windows of 400 columns each, with a step size of 50 columns. If a the block contains no more than 400 columns, the whole block is fed to RNAz. The perl programs RNAzwindow and RNAzCluster in the RNAz package are used to process the sliding window approach.

Using P > 0.5 as the threshold of confidence, RNAz predicted many ncRNAs in the *Arabidopsis *genome. When these predictions overlap, they are combined into one ncRNA.

### Computational assessment of the performance

#### Sensitivity

In order to evaluate the sensitivity of our pipeline, the known ncRNAs of *Arabidopsis *contained in miRBase and EMBL were used to compose the test dataset. The dataset contains a total of 1385 ncRNAs, including 184 microRNAs from miRBase and 638 tRNAs and 563 ncRNAs from the EMBL annotation. The sensitivity was estimated for each step of the pipeline. Among the 1385 ncRNAs, 311 are located in the expressed segments of the tiling array experiments of Group I. By using BLASTN search against the intergenic regions of rice, poplar and grape with the e-value cutoff 10^-5^, 48, 67 and 52 of the 311 ncRNAs have HSPs, respectively. Here, the e-value cutoff is purposely set more stringently than what is used in the pipeline. This is because an e-value is proportional to the length of the query sequence and the known ncRNA sequences used here are much shorter than the intergenic sequences used in the prediction pipeline. There are 41 ncRNAs conserved in ARP and 44 ncRNAs conserved in AGP. Among these ncRNAs, 30 and 29 were predicted as ncRNAs by our pipeline for ARP and AGP, respectively. There are 34 ncRNAs conserved in all four species of ARGP. Among them, 29 are contained in the intersection of the prediction results for ARP and AGP, giving rise to a sensitivity of 85.29%.

### Specificity

To estimate the specificity of our predictions, we adopted the approach used in similar works on *Plasmodium falciparum *[39] and human [7]. Our estimation was based on the triplet APP, which involved the largest number of blocks (multiple alignments). The negative samples were constructed by randomly shuffling the columns in the multiple alignments that were predicted as ncRNAs by RNAz. In the shuffling method, two shuffled columns are required to have strictly the same degree of conservation. That is, the mean pairwise identity (MPI) of each column is calculated and only columns of the same MPI value are swaped [39]. Using the cutoff P > 0.5, these randomly shuffled data gave rise a false discovery rate of 42.39%. This rate is a bit high partially because of the stringent requirement on conversation in the shuffled columns and also (perhaps more importantly) the fact that many different blocks may cover a same locus on *Arabidopsis*. Observe that the above false positive rate does not take into account of the many further analysis steps that we performed after RNAz prediction. Thus, the false positive rate in our final prediction result of novel ncRNAs is likely to be much lower.

## Competing interests

The authors declare that they have no competing interests.

## Authors' contributions

D. Song and Y. Yang designed the system and performed the computational tasks. B. Yu conducted the verification wet-bench experiments. B. Yu, B. Zheng and X. Chen analyzed the experimental results. D. Song, Y. Yang, X. Chen and T. Jiang drafted the manuscript. X. Chen and T. Jiang supervised the project. All authors read and approved the final manuscript.

## References

[B1] Plath K, Fang J, Mlynarczyk-Evans S, Cao R, Worringer K, Wang H, de la Cruz C, Otte A, Panning B, Zhang Y (2003). Role of Histone H3 Lysine 27 Methylation in X Inactivation.

[B2] Rinn J, Kertesz M, Wang J, Squazzo S, Xu X, Brugmann S, Goodnough L, Helms J, Farnham P, Segal E (2007). Functional Demarcation of Active and Silent Chromatin Domains in Human HOX Loci by Noncoding RNAs. Cell.

[B3] Yamada K, Lim J, Dale J, Chen H, Shinn P, Palm C, Southwick A, Wu H, Kim C, Nguyen M (2003). Empirical Analysis of Transcriptional Activity in the Arabidopsis Genome. Science.

[B4] Stolc V, Samanta M, Tongprasit W, Sethi H, Liang S, Nelson D, Hegeman A, Nelson C, Rancour D, Bednarek S (2005). Identification of transcribed sequences in Arabidopsis thaliana by using high-resolution genome tiling arrays. Proceedings of the National Academy of Sciences.

[B5] Zhang Z, Pang A, Gerstein M (2007). Comparative analysis of genome tiling array data reveals many novel primate-specific functional RNAs in human. BMC Evolutionary Biology.

[B6] MacIntosh G, Wilkerson C, Green P (2001). Identification and Analysis of Arabidopsis Expressed Sequence Tags Characteristic of Non-Coding RNAs. Plant Physiology.

[B7] Washietl S, Hofacker I, Lukasser M, HÄuttenhofer A, Stadler P (2005). Mapping of conserved RNA secondary structures predicts thousands of functional noncoding RNAs in the human genome. Nature Biotechnology.

[B8] Griffiths-Jones S, Moxon S, Marshall M, Khanna A, Eddy SR, Bateman A (2005). Rfam: annotating non-coding RNAs in complete genomes. Nucleic Acids Res.

[B9] Griffiths-Jones S, Grocock RJ, van Dongen S, Bateman A, Enright AJ (2006). miRBase: microRNA sequences, targets and gene nomenclature. Nucleic Acids Res.

[B10] Brown J, Echeverria M, Qu L, Lowe T, Bachellerie J, HÄuttenhofer A, Kastenmayer J, Green P, Shaw P, Marshall D (2003). Plant snoRNA database. Nucleic Acids Res.

[B11] Eddy S, Durbin R (1994). RNA sequence analysis using covariance models. Nucleic Acids Res.

[B12] Yoon B, Vaidyanthan P (2005). An overview of the role of context-sensitive HMMs in the prediction of ncRNA genes. Proc IEEE Workshop on Statistical Signal Processing, Bordeaux, France.

[B13] Will S, Reiche K, Hofacker I, Stadler P, Backofen R (2007). Inferring noncoding RNA families and classes by means of genome-scale structure-based clustering. PLoS Comput Biol.

[B14] Adai A, Johnson C, Mlotshwa S, Archer-Evans S, Manocha V, Vance V, Sundaresan V (2005). Computational prediction of miRNAs in Arabidopsis thaliana. Genome Research.

[B15] Torarinsson E, Yao Z, Wiklund E, Bramsen J, Hansen C, Kjems J, Tommerup N, Ruzzo W, Gorodkin J (2008). Comparative genomics beyond sequence-based alignments: RNA structures in the ENCODE regions. Genome Research.

[B16] McCutcheon J, Eddy S (2003). Computational identification of non-coding RNAs in Saccharomyces cerevisiae by comparative genomics. Nucleic Acids Research.

[B17] Rivas E, Eddy S (2001). Noncoding RNA gene detection using comparative sequence analysis. BMC Bioinformatics.

[B18] Axmann I, Kensche P, Vogel J, Kohl S, Herzel H, Hess W (2005). Identification of cyanobacterial non-coding RNAs by comparative genome analysis. Genome Biol.

[B19] Weinberg Z, Barrick J, Yao Z, Roth A, Kim J, Gore J, Wang J, Lee E, Block K, Sudarsan N (2007). Identification of 22 candidate structured RNAs in bacteria using the CMfinder comparative genomics pipeline. Nucleic Acids Research.

[B20] Chekanova J, Gregory B, Reverdatto S, Chen H, Kumar R, Hooker T, Yazaki J, Li P, Skiba N, Peng Q (2007). Genome-Wide High-Resolution Mapping of Exosome Substrates Reveals Hidden Features in the Arabidopsis Transcriptome. Cell.

[B21] TAIR. http://www.arabidopsis.org.

[B22] Griffiths-Jones S, Saini HK, van Dongen S, Enright AJ (2008). miRBase: tools for microRNA genomics. Nucleic Acids Research.

[B23] Griffiths-Jones S (2004). The microRNA Registry. Nucleic Acids Research.

[B24] EMBL. http://www.ebi.ac.uk/GenomeReviews/files/cellular/.

[B25] Rymarquis L, Kastenmayer J, Hüttenhofer A, Green P (2008). Diamonds in the rough: mRNA-like non-coding RNAs. Trends in Plant Science.

[B26] Eddy SR (2002). A memory-efficient dynamic programming algorithm for optimal alignment of a sequence to an RNA secondary structure. BMC Bioinformatics.

[B27] Tatusov T, Tatusov R ORF Finder (Open Reading Frame Finder). Software.

[B28] Sokal R, Rohlf F (1995). Biometry: The Principles and Practice of Statistics in Biological Research (ed.).

[B29] ASRP database. http://asrp.cgrb.oregonstate.edu/.

[B30] JY (2002). A draft sequence of the rice genome (Oryza sativa L. ssp. indica). Science.

[B31] Rice Genome Annotation Project. http://rice.plantbiology.msu.edu.

[B32] Tuskan G, DiFazio S, Jansson S, Bohlmann J, Grigoriev I, Hellsten U, Putnam N, Ralph S, Rombauts S, Salamov A (2006). The Genome of Black Cottonwood, Populus trichocarpa (Torr. & Gray). Science.

[B33] Jaillon O, Aury J, Noel B, Policriti A, Clepet C, Casagrande A, Choisne N, Aubourg S, Vitulo N, Jubin C (2007). The grapevine genome sequence suggests ancestral hexaploidization in major angiosperm phyla. Nature.

[B34] Ming R, Hou S, Feng Y, Yu Q, Dionne-Laporte A, Saw J, Senin P, Wang W, Ly B, Lewis K (2008). The draft genome of the transgenic tropical fruit tree papaya (Carica papaya Linnaeus). Nature.

[B35] Thompson J, Higgins D, Gibson T (1994). CLUSTAL W: improving the sensitivity of progressive multiple sequence alignment through sequence weighting, position-specific gap penalties and weight matrix choice. Nucleic Acids Res.

[B36] Washietl S, Hofacker I, Stadler P (2005). From The Cover: Fast and reliable prediction of noncoding RNAs. Proceedings of the National Academy of Sciences.

[B37] Missal K, Rose D, Stadler PF (2005). Non-coding RNAs in Ciona intestinalis. ECCB/JBI (Supplement of Bioinformatics).

[B38] Mourier T, Carret C, Kyes K, Christodoulou Z, Gardner P, Jeffares D, Pinches R, Barrell B, Berriman M, Griffiths-Jones S, Ivens A, Newbold C, Pain A (2008). Genome wide discovery and verification of novel structured RNAs in Plasmodium falciparum. Genome Research.

[B39] Washietl S, Hofacker IL (2004). Consensus folding of aligned sequences as a new measure for the detection of functional RNAs by comparative genomics. J Mol Biol.

